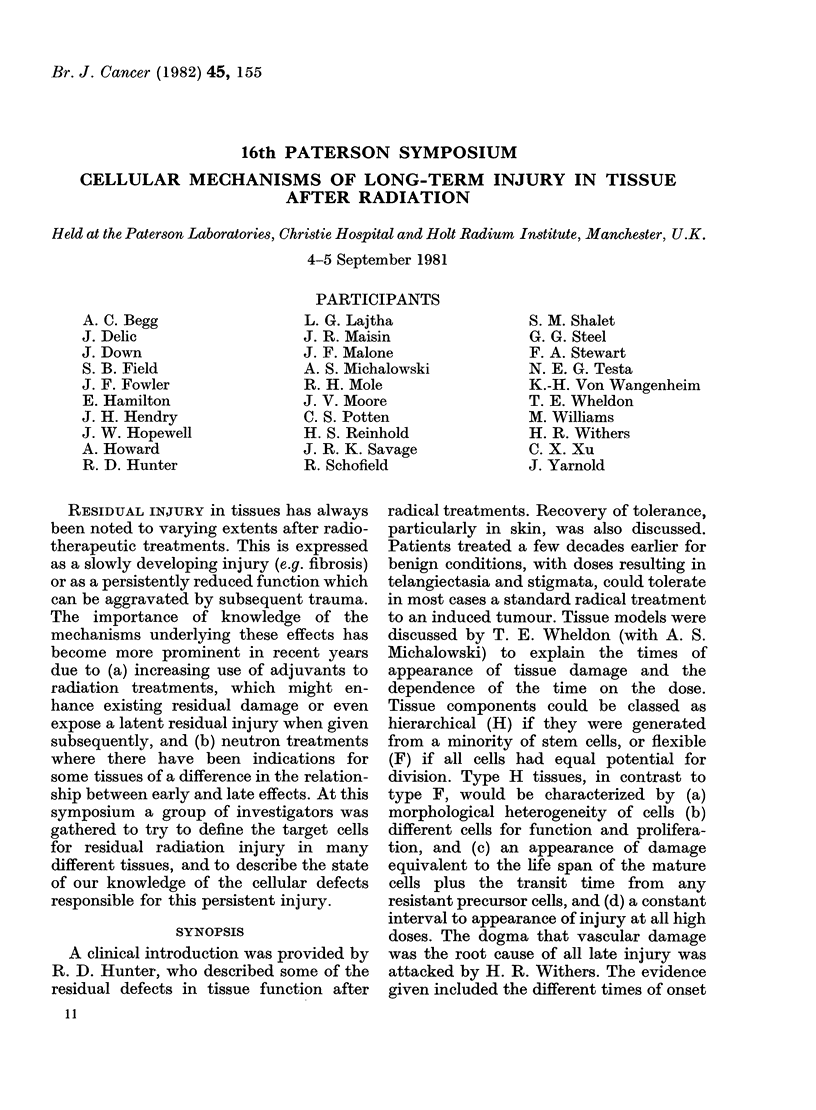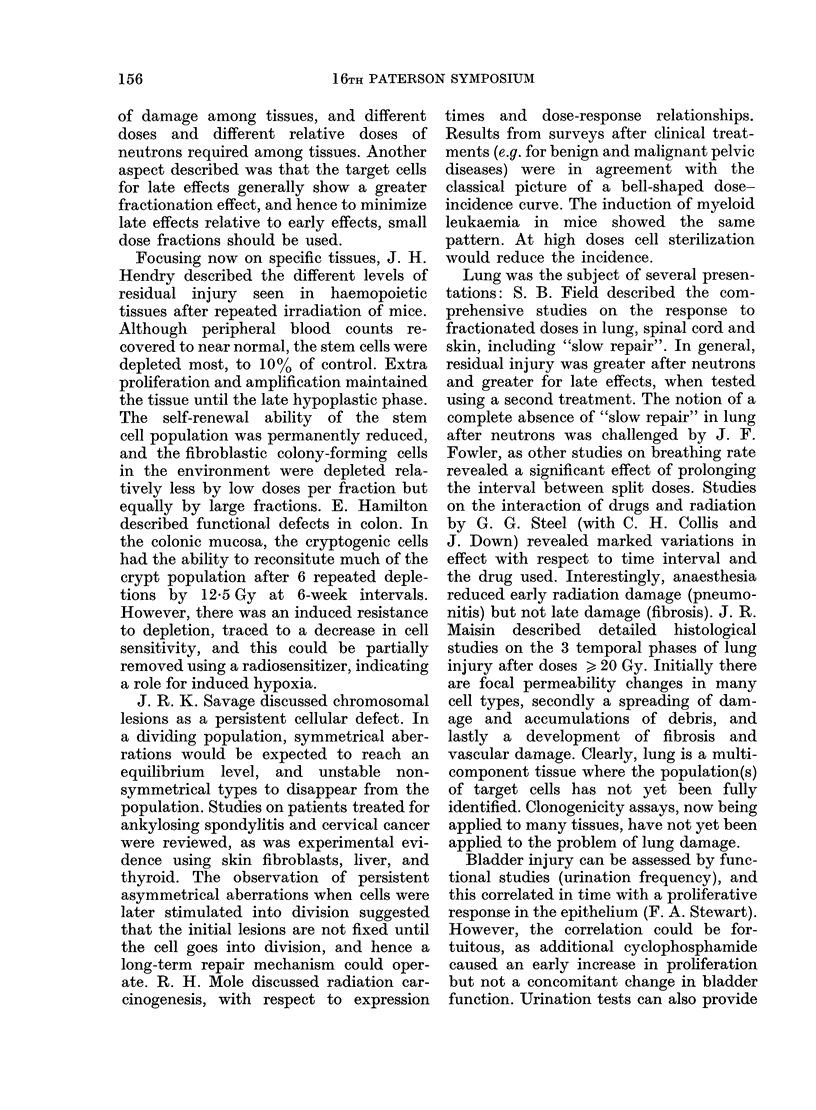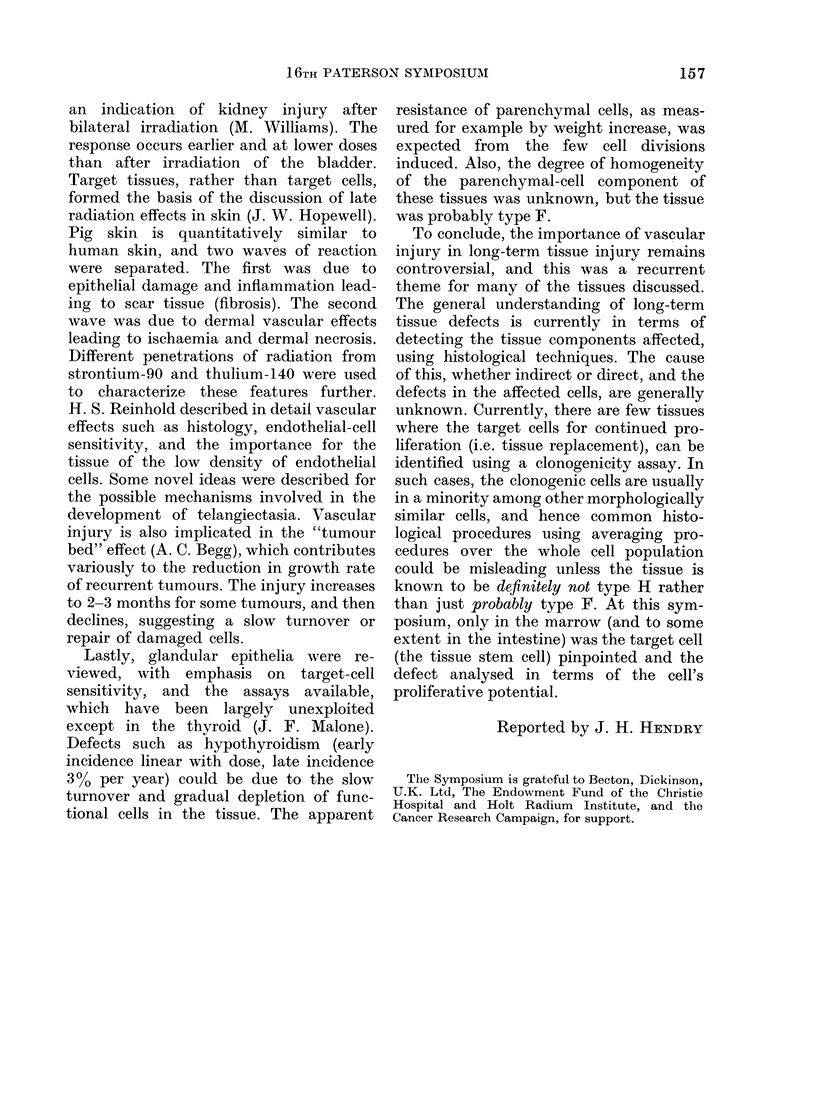# 16th Paterson Symposium—Cellular mechanisms of long-term injury in tissue after radiation

**Published:** 1982-01

**Authors:** 


*Br. J. Cancer* (1982) **45**, 155

### 16th PATERSON SYMPOSIUM

### CELLULAR MECHANISMS OF LONG-TERM INJURY IN TISSUE AFTER RADIATION

*Held at the Paterson Laboratories, Christie Hospital and Holt Radium Institute, Manchester, U.K.*

4–5 September 1981

### PARTICIPANTS

| | | |
|---|---|---|
| A. C. Begg | L. G. Lajtha | S. M. Shalet |
| J. Delic | J. R. Maisin | G. G. Steel |
| J. Down | J. F. Malone | F. A. Stewart |
| S. B. Field | A. S. Michalowski | N. E. G. Testa |
| J. F. Fowler | R. H. Mole | K.-H. Von Wangenheim |
| E. Hamilton | J. V. Moore | T. E. Wheldon |
| J. H. Hendry | C. S. Potten | M. Williams |
| J. W. Hopewell | H. S. Reinhold | H. R. Withers |
| A. Howard | J. R. K. Savage | C. X. Xu |
| R. D. Hunter | R. Schofield | J. Yarnold |

Residual injury in tissues has always been noted to varying extents after radiotherapeutic treatments. This is expressed as a slowly developing injury (*e.g.* fibrosis) or as a persistently reduced function which can be aggravated by subsequent trauma. The importance of knowledge of the mechanisms underlying these effects has become more prominent in recent years due to (a) increasing use of adjuvants to radiation treatments, which might enhance existing residual damage or even expose a latent residual injury when given subsequently, and (b) neutron treatments where there have been indications for some tissues of a difference in the relationship between early and late effects. At this symposium a group of investigators was gathered to try to define the target cells for residual radiation injury in many different tissues, and to describe the state of our knowledge of the cellular defects responsible for this persistent injury.

### SYNOPSIS

A clinical introduction was provided by R. D. Hunter, who described some of the residual defects in tissue function after radical treatments. Recovery of tolerance, particularly in skin, was also discussed. Patients treated a few decades earlier for benign conditions, with doses resulting in telangiectasia and stigmata, could tolerate in most cases a standard radical treatment to an induced tumour. Tissue models were discussed by T. E. Wheldon (with A. S. Michalowski) to explain the times of appearance of tissue damage and the dependence of the time on the dose. Tissue components could be classed as hierarchical (H) if they were generated from a minority of stem cells, or flexible (F) if all cells had equal potential for division. Type H tissues, in contrast to type F, would be characterized by (a) morphological heterogeneity of cells (b) different cells for function and proliferation, and (c) an appearance of damage equivalent to the life span of the mature cells plus the transit time from any resistant precursor cells, and (d) a constant interval to appearance of injury at all high doses. The dogma that vascular damage was the root cause of all late injury was attacked by H. R. Withers. The evidence given included the different times of onset of damage among tissues, and different doses and different relative doses of neutrons required among tissues. Another aspect described was that the target cells for late effects generally show a greater fractionation effect, and hence to minimize late effects relative to early effects, small dose fractions should be used.

Focusing now on specific tissues, J. H. Hendry described the different levels of residual injury seen in haemopoietic tissues after repeated irradiation of mice. Although peripheral blood counts recovered to near normal, the stem cells were depleted most, to 10% of control. Extra proliferation and amplification maintained the tissue until the late hypoplastic phase. The self-renewal ability of the stem cell population was permanently reduced, and the fibroblastic colony-forming cells in the environment were depleted relatively less by low doses per fraction but equally by large fractions. E. Hamilton described functional defects in colon. In the colonic mucosa, the cryptogenic cells had the ability to reconsitute much of the crypt population after 6 repeated depletions by 12·5 Gy at 6-week intervals. However, there was an induced resistance to depletion, traced to a decrease in cell sensitivity, and this could be partially removed using a radiosensitizer, indicating a role for induced hypoxia.

J. R. K. Savage discussed chromosomal lesions as a persistent cellular defect. In a dividing population, symmetrical aberrations would be expected to reach an equilibrium level, and unstable non-symmetrical types to disappear from the population. Studies on patients treated for ankylosing spondylitis and cervical cancer were reviewed, as was experimental evidence using skin fibroblasts, liver, and thyroid. The observation of persistent asymmetrical aberrations when cells were later stimulated into division suggested that the initial lesions are not fixed until the cell goes into division, and hence a long-term repair mechanism could operate. R. H. Mole discussed radiation carcinogenesis, with respect to expression times and dose-response relationships. Results from surveys after clinical treatments (*e.g.* for benign and malignant pelvic diseases) were in agreement with the classical picture of a bell-shaped dose–incidence curve. The induction of myeloid leukaemia in mice showed the same pattern. At high doses cell sterilization would reduce the incidence.

Lung was the subject of several presentations: S. B. Field described the comprehensive studies on the response to fractionated doses in lung, spinal cord and skin, including "slow repair". In general, residual injury was greater after neutrons and greater for late effects, when tested using a second treatment. The notion of a complete absence of "slow repair" in lung after neutrons was challenged by J. F. Fowler, as other studies on breathing rate revealed a significant effect of prolonging the interval between split doses. Studies on the interaction of drugs and radiation by G. G. Steel (with C. H. Collis and J. Down) revealed marked variations in effect with respect to time interval and the drug used. Interestingly, anaesthesia reduced early radiation damage (pneumonitis) but not late damage (fibrosis). J. R. Maisin described detailed histological studies on the 3 temporal phases of lung injury after doses $\geqslant 20$ Gy. Initially there are focal permeability changes in many cell types, secondly a spreading of damage and accumulations of debris, and lastly a development of fibrosis and vascular damage. Clearly, lung is a multicomponent tissue where the population(s) of target cells has not yet been fully identified. Clonogenicity assays, now being applied to many tissues, have not yet been applied to the problem of lung damage.

Bladder injury can be assessed by functional studies (urination frequency), and this correlated in time with a proliferative response in the epithelium (F. A. Stewart). However, the correlation could be fortuitous, as additional cyclophosphamide caused an early increase in proliferation but not a concomitant change in bladder function. Urination tests can also provide an indication of kidney injury after bilateral irradiation (M. Williams). The response occurs earlier and at lower doses than after irradiation of the bladder. Target tissues, rather than target cells, formed the basis of the discussion of late radiation effects in skin (J. W. Hopewell). Pig skin is quantitatively similar to human skin, and two waves of reaction were separated. The first was due to epithelial damage and inflammation leading to scar tissue (fibrosis). The second wave was due to dermal vascular effects leading to ischaemia and dermal necrosis. Different penetrations of radiation from strontium-90 and thulium-140 were used to characterize these features further. H. S. Reinhold described in detail vascular effects such as histology, endothelial-cell sensitivity, and the importance for the tissue of the low density of endothelial cells. Some novel ideas were described for the possible mechanisms involved in the development of telangiectasia. Vascular injury is also implicated in the "tumour bed" effect (A. C. Begg), which contributes variously to the reduction in growth rate of recurrent tumours. The injury increases to 2–3 months for some tumours, and then declines, suggesting a slow turnover or repair of damaged cells.

Lastly, glandular epithelia were reviewed, with emphasis on target-cell sensitivity, and the assays available, which have been largely unexploited except in the thyroid (J. F. Malone). Defects such as hypothyroidism (early incidence linear with dose, late incidence 3% per year) could be due to the slow turnover and gradual depletion of functional cells in the tissue. The apparent resistance of parenchymal cells, as measured for example by weight increase, was expected from the few cell divisions induced. Also, the degree of homogeneity of the parenchymal-cell component of these tissues was unknown, but the tissue was probably type F.

To conclude, the importance of vascular injury in long-term tissue injury remains controversial, and this was a recurrent theme for many of the tissues discussed. The general understanding of long-term tissue defects is currently in terms of detecting the tissue components affected, using histological techniques. The cause of this, whether indirect or direct, and the defects in the affected cells, are generally unknown. Currently, there are few tissues where the target cells for continued proliferation (i.e. tissue replacement), can be identified using a clonogenicity assay. In such cases, the clonogenic cells are usually in a minority among other morphologically similar cells, and hence common histological procedures using averaging procedures over the whole cell population could be misleading unless the tissue is known to be *definitely not* type H rather than just *probably* type F. At this symposium, only in the marrow (and to some extent in the intestine) was the target cell (the tissue stem cell) pinpointed and the defect analysed in terms of the cell's proliferative potential.

Reported by J. H. HENDRY

The Symposium is grateful to Becton, Dickinson, U.K. Ltd, The Endowment Fund of the Christie Hospital and Holt Radium Institute, and the Cancer Research Campaign, for support.